# Retinal microvasculature observations of fellow eyes after intra-arterial chemotherapy for unilateral retinoblastoma using optical coherence tomography angiography

**DOI:** 10.3389/fmed.2022.1015301

**Published:** 2023-01-10

**Authors:** Yijing Chen, Jianbo Mao, Ziyi Xiang, Zhengxi Zhang, Shian Zhang, Sulan Wu, Lijun Shen

**Affiliations:** ^1^Department of Ophthalmology, Center for Rehabilitation Medicine, Affiliated People’s Hospital, Hangzhou Medical College, Hangzhou, Zhejiang, China; ^2^Department of Retina Center, Affiliated Eye Hospital of Wenzhou Medical University, Hangzhou, Zhejiang, China

**Keywords:** retinoblastoma, intra-arterial chemotherapy, optical coherence tomography angiography, microvasculature, retina

## Abstract

**Purpose:**

To investigate the characteristics of the retinal microvasculature of the fellow eyes in patients with unilateral retinoblastoma (RB) after intra-arterial chemotherapy (IAC) through optical coherence tomography angiography.

**Methods:**

This retrospective study enrolled 11 fellow eyes of patients with unilateral RB receiving IAC (group I), nine fellow eyes of patients with unilateral RB receiving IAC and intravenous chemotherapy (IVC) (group II), and 14 age-matched normal eyes (control group). Optical coherence tomography angiography was performed on all individuals. Vascular density of superficial capillary plexus and deep capillary plexus (DCP), foveal avascular zone related parameters, and retinal thickness were measured and compared among the three groups.

**Results:**

There was no statistical difference in age and logMAR visual acuity among the three groups. Compared with the control group, the vascular density of the DCP was lower in group I and II. Decreased vascular density of FD-300 and thinner thickness of outer plexus layer to Bruch’s membrane were detected in group II compared with the control group. The vascular density and retinal thickness showed no differences between group I and II.

**Conclusion:**

The decreased vascular density in the DCP without measurable visual impairment was observed in fellow eyes after IAC or IAC + IVC for unilateral RB. Further studies with a larger sample would be necessary to determine the clinical significance of these findings.

## Introduction

Retinoblastoma (RB) is an aggressive intraocular malignant tumor that originates from the retina in children (1). Chemotherapy is the most commonly used treatment for RB. Intravenous chemotherapy (IVC) reduces the volume of the tumor effectively through systemic chemotherapy such as vincristine, etoposide, and carboplatin. However, it is often accompanied by systemic toxicities such as nausea, vomiting, fever, bone marrow suppression, transient hemocytopenia, hearing loss, and acute leukemia (2–5). Recently, superselective intra-arterial chemotherapy (IAC) has become the primary treatment for retinoblastoma as it has fewer systemic complications compared with IVC. It superselectively delivers chemotherapeutic drugs to the ophthalmic artery (OA) through the femoral artery or internal carotid artery using a microcatheter so as to achieve and limit the highest concentration of chemotherapeutic drugs in the tumor area to exert the maximum effect, and has little influence on other tissues and organs (6). However, vascular complications including choroidal ischemia or retinal artery embolization have been observed in eyes receiving IAC (7). But these studies focused on the microvascular changes of treated eyes. For patients with unilateral retinoblastoma, it is essential to investigate the safety of the fellow eyes after IAC treatment.

Nowadays, optical coherence tomography angiography (OCTA) can non-invasively observe and quantify the structure of the retina. It plays a significant role in the detection and follow-up of various retinal diseases. It has also been used to investigate the microvasculature effects of intraocular tumors (8, 9). For RB, changes in microvasculature before and after treatments have been evaluated by OCTA (7, 10). Thomas et al. reported persistently congested donor vessels or increasing vessel density over time in OCTA may indicated persistent disease activity or tumor reactivation (11). Yi et al. suggested that a slight reduction in retinal thickness and the deep foveal retinal vascular density seemed to occur in bilateral RB eyes treated with IVC (12). A slight reduction in deep capillary density was detected both in eyes with bilateral RB and in fellow eyes with unilateral retinoblastoma after IVC *via* OCTA (13). However, there are limited reports in the literature about microvascular changes of OCTA after IAC.

In this study, the retinal microvasculature in fellow eyes with unilateral retinoblastoma treated with IAC was observed and evaluated using OCTA, to understand the potential ocular toxicities in the pediatric population.

## Materials and methods

### Ethics statement and consent to participate

This retrospective study adhered to the declaration of Helsinki. Informed consent was obtained from all subjects involved in the study, and the protocol was approved by the ethics review board of Wenzhou Medical University Affiliated Eye Hospital.

### Study participants

This study enrolled 20 individuals who were affected by unilateral retinoblastoma that had a normal fellow eye. They had completed chemotherapy in early life and underwent a regularly scheduled examination under anesthesia in Wenzhou Medical University Affiliated Eye Hospital from August 2020 to November 2021. This study divided the participators into two groups: unilateral RB patients treated with IAC (group I, *n* = 11) and unilateral RB patients treated with IAC + IVC (group II, *n* = 9). Patients who were affected by extraocular tumor other than retinoblastoma were excluded. Any patient who received additional treatment of plaque radiotherapy, external beam radiotherapy, subconjunctival chemotherapy, or intravitreal chemotherapy was excluded from the analysis. Patients with side effects including optic nerve swelling, retinal detachment, bleeding, OA spasm, loss of blood supply to the eye, or irreversible vision loss were excluded. Fellow eyes with any abnormal symptoms were also excluded. Age-matched healthy children were included as the controls (control group, *n* = 14). The individuals in the control group were healthy with no ocular disorders or known systemic diseases. One eye of them was randomly selected for inclusion in this study.

### Ophthalmological examination

All the children had a review of medical history which included the age at the time of this study, sex, family history, modality of treatment used, the number of IVC or IAC cycles, and interval time from the last chemotherapy to time of OCTA imaging. All patients underwent comprehensive ophthalmologic examinations which included best-corrected visual acuity (BCVA), refraction measurement by auto refraction, spherical equivalence (SE) calculation, slit-lamp biomicroscopy, Doppler B ultrasound and wild-field fundus imaging. Wide-field fundus imaging was conducted using the Optos 200Tx (Optos, Marlborough, MA, USA).

### Optical coherence tomography angiography

Optical coherence tomography angiography images were acquired by the RTVue XR Avanti AngioVue (Optovue, Inc., Fremont, CA, USA). The examination was performed by the same skilled doctor. Angio retina 3 mm × 3 mm procedure was selected, covering the square area with 3 mm centered on the fovea. The software automatically divided the zone into an inner and an outer ring with a diameter of 1 and 3 mm, respectively centered on the fovea, defining the fovea as the area within the 1 mm diameter ring, the parafovea as a band area with a width of 2 mm between the inner and the outer ring. The whole image of the circle area with 3 mm and the parafovea was divided into the superior-hemi and the inferior-hemi, respectively. Four rays further divided the parafovea into four areas—the superior, the temporal, the inferior, and the nasal.

The device used the crossover amplitude decorrelation blood flow imaging algorithm split-spectrum amplitude-decorrelation angiography (SSADA) to quantify the retina vessel density of the retina, foveal avascular zone (FAZ) area, FAZ perimeter (PERIM), acircularity index (AI), and foveal vessel density in a 300-μm-wide region around FAZ (FD-300). The superficial capillary plexus (SCP) measurement area was defined as the internal limiting membrane (ILM) to the inner plexus layer (IPL) −10 μm, and the deep capillary plexus (DCP) measurement area was defined as the IPL −10 μm to the outer plexus layer (OPL) +10 μm. The vessel density was defined as the percentage of vessel cavity area in the sample area automatically calculated by the software. Retinal layer segmentation was performed automatically by built-in AngioVue software. The distance of the ILM to the retinal pigment epithelium (the full layer), the ILM to the IPL (the inner layer), the inner nuclear layer to the retinal pigment epithelium (the outer layer), the internal limiting membrane to Bruch’s membrane (ILM-BRM), the outer plexus layer to Bruch’s membrane (OPL-BRM), the ellipsoid zone to Bruch’s membrane (ISOS-BRM) of the retina were divided by 5 parts: the fovea and the superior, the temporal, the inferior, the nasal of the parafovea. The distance of the full layer was further divided as shown in Table 4. All scans were reviewed by the same researcher and were required for high image quality (signal strength ≥ 7/10), accurate segmentation, and absence of major artifacts.

**TABLE 1:** The demographic and clinical data of the patients with unilateral retinoblastoma and healthy controls.

	Group I	Group II	Control	*P*-value		
	(*n* = 11)	(*n* = 9)	(*n* = 14)	I vs. II	I vs. C	II vs. C
Age (years)	6.17 ± 1.41	5.86 ± 2.41	6.92 ± 1.32	0.684	0.280	0.152
**Gender**						
Male	5	8	5	0.070	0.697	0.029*
Female	6	1	9			
**Eyes**						
OD	4	5	6	0.653	1.000	0.680
OS	7	4	8			
IVC times	–	3.22 ± 2.73	–	–	–	–
IAC times	3.64 ± 1.29	3.67 ± 1.32	–	0.882	–	–
Duration from last chemotherapy to OCTA (years)	3.91 ± 1.43	2.88 ± 1.43	–	0.244	–	–
BCVA (logMAR)	0	0	0	1.000	1.000	1.000
Family history	0	0	0	–	–	–

OD, oculus dextrus; OS, oculus sinister; IVC, intravenous chemotherapy; IAC, intra-arterial chemotherapy; BCVA, best-corrected visual acuity.

*P < 0.05.

**TABLE 2:** Vessel densities and retinal thicknesses of three groups.

		Group I	Group II	Control	*P*-value		
		(*n* = 11)	(*n* = 9)	(*n* = 14)	I vs. II	I vs. C	II vs. C
**FAZ**							
	Area (mm^2^)	0.27 ± 0.13	0.22 ± 0.09	0.27 ± 0.11	0.394	0.951	0.330
	PERIM (mm)	1.98 ± 0.59	1.90 ± 0.37	2.02 ± 0.40	0.698	0.830	0.533
	AI	1.12 ± 0.02	1.17 ± 0.08	1.12 ± 0.04	0.075	0.943	0.065
	FD-300 (%)	50.77 ± 3.12	48.21 ± 2.53	51.37 ± 3.48	0.087	0.647	0.025*
**Vascular density (%)**							
SCP	Whole image	48.36 ± 1.61	48.73 ± 2.45	47.97 ± 1.31	0.635	0.593	0.319
	Superior-hemi	48.39 ± 1.75	48.71 ± 2.71	48.51 ± 1.85	0.734	0.890	0.819
	Inferior-hemi	48.29 ± 1.74	48.71 ± 2.20	47.26 ± 1.85	0.628	0.192	0.086
	Fovea	22.93 ± 10.28	24.53 ± 6.91	20.76 ± 6.34	0.656	0.503	0.275
	ParaFovea	50.51 ± 1.73	50.95 ± 2.54	50.51 ± 1.66	0.625	0.998	0.607
	PSuperior-hemi	50.41 ± 1.80	51.00 ± 3.37	51.17 ± 2.22	0.603	0.441	0.874
	PInferior-hemi	50.62 ± 2.01	50.70 ± 1.84	49.79 ± 1.88	0.925	0.288	0.272
	PTempo	50.81 ± 2.05	50.04 ± 2.60	49.36 ± 2.28	0.473	0.125	0.507
	PSuperior	51.06 ± 1.76	51.75 ± 3.30	52.32 ± 2.62	0.568	0.232	0.618
	PNasal	48.81 ± 3.15	50.66 ± 3.49	49.31 ± 2.02	0.158	0.662	0.277
	PInferior	51.37 ± 2.13	50.88 ± 2.13	51.18 ± 2.00	0.600	0.820	0.742
DCP	Whole image	49.78 ± 4.11	49.10 ± 4.91	53.09 ± 3.08	0.704	0.047*	0.025*
	Superior-hemi	49.87 ± 4.66	49.93 ± 4.83	54.20 ± 2.74	0.973	0.012*	0.019*
	Inferior-hemi	49.74 ± 3.70	48.60 ± 4.96	51.76 ± 4.03	0.551	0.239	0.087
	Fovea	36.49 ± 8.73	37.27 ± 5.81	35.89 ± 7.69	0.822	0.847	0.676
	ParaFovea	51.94 ± 4.87	51.69 ± 5.65	55.35 ± 2.79	0.902	0.060	0.066
	PSuperior-hemi	51.97 ± 5.23	52.04 ± 5.82	56.57 ± 2.44	0.975	0.015*	0.028*
	PInferior-hemi	51.89 ± 4.61	50.70 ± 5.58	54.03 ± 3.70	0.564	0.252	0.096
	PTempo	52.67 ± 4.79	51.74 ± 5.23	54.89 ± 3.06	0.639	0.205	0.105
	PSuperior	51.20 ± 5.43	51.19 ± 7.09	56.61 ± 2.80	0.996	0.012*	0.020*
	PNasal	52.55 ± 5.05	51.64 ± 5.98	55.86 ± 2.80	0.663	0.081	0.038*
	PInferior	51.26 ± 4.96	50.62 ± 6.01	53.85 ± 4.34	0.782	0.218	0.149
**Thickness of the fovea (μm)**							
	Full	246.45 ± 18.10	241.67 ± 13.24	245.43 ± 15.77	0.517	0.642	0.560
	Inner	49.73 ± 12.52	49.22 ± 8.27	47.86 ± 7.17	0.919	0.881	0.679
	Outer	201.09 ± 9.24	196.56 ± 7.65	203.71 ± 10.70	0.254	0.525	0.097
	ILM-BRM	256.00 ± 17.96	251.00 ± 12.81	256.36 ± 15.04	0.493	0.687	0.179
	OPL-BRM	168.18 ± 10.33	159.22 ± 10.51	169.57 ± 9.65	0.057	0.735	0.023*
	ISOS-BRM	57.82 ± 1.72	58.56 ± 2.07	59.00 ± 1.92	0.331	0.222	0.975

FAZ, foveal avascular zone; PERIM, FAZ perimeter; AI, acircularity index; FD-300, foveal vessel density in a 300-μm-wide region around FAZ; SCP, superficial capillary plexus; DCP, deep capillary plexus.

*P < 0.05.

**TABLE 3:** The ratio of SCP/DCP in vascular density of three groups.

		Group I	Group II	Control	*P*-value		
		(*n* = 11)	(*n* = 9)	(*n* = 14)	I vs. II	I vs. C	II vs. C
SCP/DCP in VD	Whole image	0.98 ± 0.09	1.00 ± 0.14	0.91 ± 0.06	0.524	0.072	0.021*
	Superior-hemi	0.98 ± 0.10	0.99 ± 0.14	0.90 ± 0.05	0.847	0.045*	0.038*
	Inferior-hemi	0.98 ± 0.08	1.02 ± 0.14	0.92 ± 0.06	0.361	0.134	0.021*
	Fovea	0.60 ± 0.13	0.65 ± 0.10	0.57 ± 0.08	0.336	0.407	0.078
	ParaFovea	0.98 ± 0.08	1.00 ± 0.16	0.91 ± 0.06	0.640	0.114	0.058
	PSuperior-hemi	0.98 ± 0.09	1.00 ± 0.17	0.91 ± 0.05	0.708	0.096	0.060
	PInferior-hemi	0.98 ± 0.08	1.01 ± 0.14	0.93 ± 0.07	0.462	0.160	0.040*
	PTempo	0.97 ± 0.10	0.98 ± 0.14	0.90 ± 0.05	0.884	0.079	0.081
	PSuperior	1.01 ± 0.09	1.03 ± 0.19	0.93 ± 0.06	0.618	0.098	0.046*
	PNasal	0.93 ± 0.05	1.00 ± 0.17	0.88 ± 0.05	0.144	0.229	0.010*
	PInferior	1.01 ± 0.09	1.02 ± 0.16	0.96 ± 0.09	0.830	0.255	0.196

VD, vascular density; SCP, superficial capillary plexus; DCP, deep capillary plexus.

*P < 0.05.

**TABLE 4:** The ratio of vascular densities/the thickness of the full layer in three groups.

		Group I	Group II	Control	*P*-value		
		(*n* = 11)	(*n* = 9)	(*n* = 14)	I vs. II	I vs. C	II vs. C
VD of SCP/the full layer	Whole image	0.153 ± 0.009	0.161 ± 0.008	0.154 ± 0.008	0.053	0.904	0.054
	Superior-hemi	0.153 ± 0.009	0.161 ± 0.009	0.155 ± 0.009	0.085	0.634	0.163
	Inferior-hemi	0.154 ± 0.009	0.160 ± 0.008	0.153 ± 0.008	0.083	0.782	0.042*
	Fovea	0.091 ± 0.035	0.101 ± 0.027	0.084 ± 0.023	0.430	0.526	0.158
	ParaFovea	0.156 ± 0.009	0.163 ± 0.008	0.157 ± 0.008	0.073	0.726	0.114
	PSuperior-hemi	0.156 ± 0.009	0.164 ± 0.009	0.159 ± 0.010	0.093	0.445	0.274
	PInferior-hemi	0.156 ± 0.009	0.164 ± 0.009	0.159 ± 0.010	0.098	0.792	0.049*
	PTempo	0.161 ± 0.009	0.164 ± 0.008	0.158 ± 0.010	0.539	0.400	0.164
	PSuperior	0.156 ± 0.010	0.164 ± 0.008	0.161 ± 0.010	0.058	0.205	0.383
	PNasal	0.150 ± 0.010	0.161 ± 0.011	0.152 ± 0.009	0.018*	0.662	0.034*
	PInferior	0.158 ± 0.010	0.163 ± 0.009	0.159 ± 0.008	0.281	0.927	0.302
VD of DCP/the full layer	Whole image	0.158 ± 0.018	0.162 ± 0.017	0.170 ± 0.014	0.582	0.069	0.243
	Superior-hemi	0.158 ± 0.019	0.166 ± 0.018	0.173 ± 0.014	0.310	0.031*	0.340
	Inferior-hemi	0.158 ± 0.017	0.160 ± 0.017	0.167 ± 0.016	0.845	0.234	0.353
	Fovea	0.147 ± 0.026	0.154 ± 0.020	0.145 ± 0.026	0.529	0.901	0.437
	ParaFovea	0.161 ± 0.019	0.166 ± 0.020	0.173 ± 0.012	0.516	0.091	0.376
	PSuperior-hemi	0.161 ± 0.020	0.167 ± 0.021	0.176 ± 0.012	0.884	0.041*	0.280
	PInferior-hemi	0.161 ± 0.019	0.163 ± 0.019	0.169 ± 0.013	0.809	0.228	0.373
	PTempo	0.168 ± 0.021	0.170 ± 0.019	0.176 ± 0.014	0.813	0.262	0.436
	PSuperior	0.156 ± 0.020	0.163 ± 0.025	0.174 ± 0.012	0.432	0.024*	0.193
	PNasal	0.162 ± 0.018	0.164 ± 0.021	0.172 ± 0.011	0.732	0.128	0.274
	PInferior	0.158 ± 0.020	0.162 ± 0.021	0.167 ± 0.015	0.641	0.250	0.539

VD, vascular density; SCP, superficial capillary plexus; DCP, deep capillary plexus.

*P < 0.05.

### Data analysis

The Statistical Package for the Social Sciences (Version 23, SPSS Inc., Chicago, IL, USA) was used for statistical analysis. Variable normality was inspected using the Shapiro–Wilk test. The Student’s *t*-test and one-way ANOVA were used to compare data with normal distributions. Comparisons of non-normal distributional data were performed using the Mann–Whitney U test and the Kruskal–Wallis H test. Categorical variables were compared by the Chi-square test. Data was statistically significant if *P* was less than 0.05.

## Results

A total of 20 individuals affected by unilateral retinoblastoma and 14 healthy controls were enrolled in this study. The demographics and clinical features were shown in Table 1. The melphalan was used in IAC while the vincristine-etoposide-carboplatin (VEC) program in IVC. The melphalan dose was 3, 5, or 7.5 mg, adjusted for the age and tumor size. VEC treatment was vincristine 1.5 mg/m^2^ per cycle, etoposide 150 mg/m^2^ per cycle, and carboplatin 560 mg/m^2^ per cycle. Recurrence was observed in three patients including two patients treated with IAC + IVC and one patient with IAC. They underwent the enucleation at last. The tumor lesions of the remaining 17 patients remained stable. The average age of group I was 6.17 ± 1.41 years old. The average age of group II was 5.86 ± 2.41 years old. In group I, the mean number of IAC treatments was 3.64 ± 1.29 (range, 3–7). In group II, the mean number of times receiving IVC and IAC treatments was 3.22 ± 2.73 (range, 1–8) and 3.67 ± 1.32 (range, 3–7), respectively. The time interval from the last chemotherapy to the OCTA measurement in group I and group II was 3.91 ± 1.43 (range 1.53–5.65) years and 2.88 ± 1.43 (range 1.26–5.13) years, respectively. There was no significant difference among the study groups for the mean logMAR visual acuity.

**FIGURE 1:**
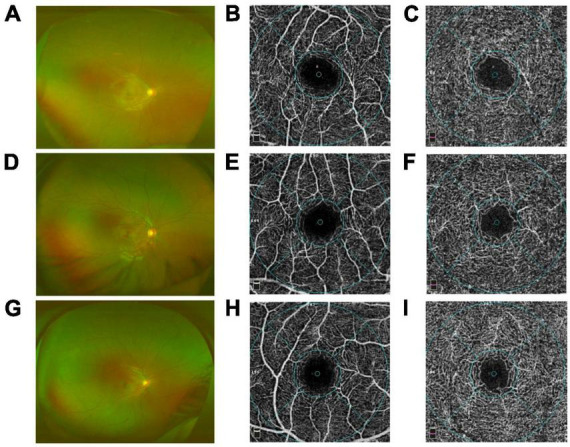
Comparison of the capillary plexuses in group I [eyes treated with intra-arterial chemotherapy (IAC)], group II [eyes treated with IAC and intravenous chemotherapy (IVC)], and the control group eyes on optical coherence tomography angiography (OCTA). Wide-field fundus images of group I **(A)**, group II **(D)**, control group **(G)** were obtained. No difference in the superficial plexus capillary density was detected between group I **(B)**, group II **(E)**, control group **(H)**. By contrast, the deep plexus capillary density decreased in group I **(C)** and group II **(F)** compared with the control group **(I)**, respectively.

**FIGURE 2:**
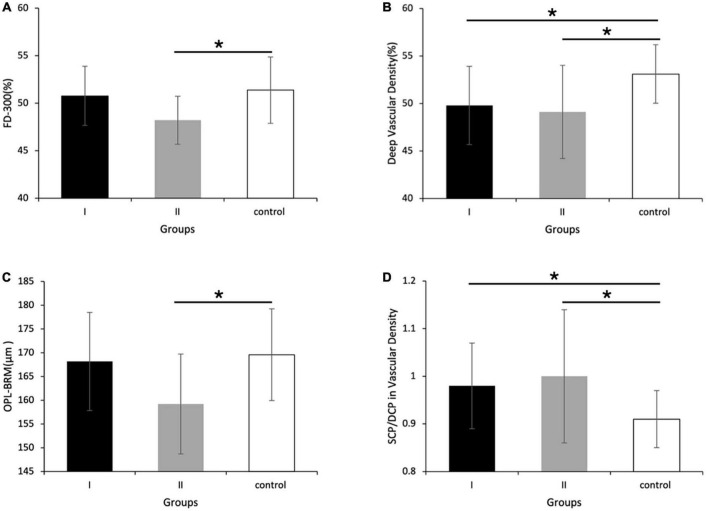
Vascular density and retina thickness in group I [eyes treated with intra-arterial chemotherapy (IAC)], group II [eyes treated with IAC and intravenous chemotherapy (IVC)], and the control group. Compared with the control group, **(A)** the FD-300 [foveal vessel density in a 300-μm-wide region around foveal avascular zone (FAZ)], **(B)** the vascular density of deep capillary plexus (DCP), and **(C)** the thickness of outer plexus layer to Bruch’s membrane (OPL-BRM) was lower in group II. **(D)** The ratio of superficial capillary plexus (SCP)/DCP in vascular density of the superior-hemi whole image was higher in group I and II, respectively compared to the control group. **(B)** The vascular density of DCP in group I was also detected to decrease. *P < 0.05.

**FIGURE 3:**
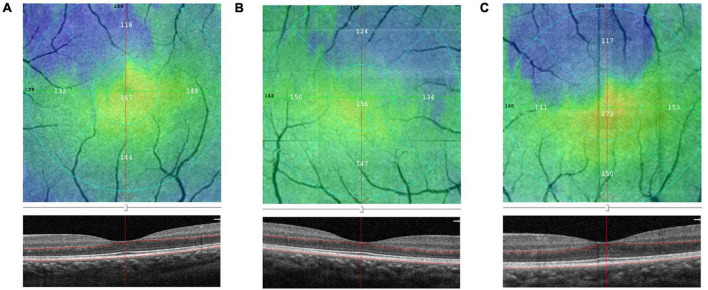
Comparison of the outer plexus layer to Bruch’s membrane (OPL-BRM) thicknesses of the fovea in group I [eyes treated with intra-arterial chemotherapy (IAC)], group II [eyes treated with IAC and intravenous chemotherapy (IVC)], and the control group eyes on optical coherence tomography angiography (OCTA). The B-scan and en-face images of group I **(A)**, group II **(B)**, control group **(C)** were obtained. The thickness of OPL-BRM in group II was detected to be reduced compared to the control group.

Compared with the control group, group I and II showed lower vascular density in DCP, respectively (Figures 1, 2B and Table 2). More specifically, the vascular density of the whole image, the superior-hemi whole image, the superior-hemi parafovea, and the superior parafovea demonstrated significant differences between group I and the control group. In group II, the deduction of vascular density was similar to group I. Moreover, there was significant difference in the nasal parafovea between group II and the control group. There was no statistical difference in the parameters related to FAZ including FAZ area, FAZ perimeter, and AI between the control group with group I or II. However, the vascular density of FD-300 in group II was lower than the control group (*P* = 0.025) (Figure 2A and Table 2). The FAZ area seemed to be smaller in group II than in the control group though the difference did not reach statistical significance. The thickness of OPL-BRM in the fovea in group II was thinner than in the control group (*P* = 0.023). While the thickness of the full layer, the outer layer, the ILM-BRM, and the ISOS-BRM in the fovea in group II tended to decrease compared to the control group, but the data did not reach statistical significance (Figures 2C, 3 and Table 2). The ratio of SCP/DCP in vascular density was detected to be higher both in group I and II when compared with the control group (Figure 2D and Table 3). For the full layer, compared to the control group, the ratio of SCP vascular density/the retina thickness at the corresponding position in group II was higher while the ratio of DCP vascular density/the retina thickness in group I was lower (Table 4). In group I, the correlation analysis between retinal microvasculature and the number of times receiving IAC was performed, showing no correlation.

Comparing group I with II, the SCP vascular density/the thickness of the full layer at the nasal parafovea in group II was higher (*P* = 0.018) (Table 4). But there were no statistically significant differences in the FAZ area, the vascular density in the superficial foveal and parafoveal area, the vascular density in the deep foveal and parafoveal area, and the thickness of the fovea between group I and II.

## Discussion

The blood supply to the inner layer of the retina and choroid layer is primarily provided by the central retinal arteries and the posterior ciliary arteries, respectively, which originate from the OA. During the process of the IAC, chemotherapeutic agents reach the focus through the vasculature mentioned above, leading to excellent efficacy. IAC has the advantages of few systematic toxicities and obvious effect on tumors due to the great ocular-to-systemic drug concentration ratio (14). As a result, complications associated with ophthalmic vascular (7) and retinal structure were common in treated eyes after receiving IAC.

In this study, we found a slight reduction in DCP vascular density of the fellow eyes in patients with unilateral retinoblastoma after receiving IAC. This was the first time that these microvasculature changes have been reported. In our study, the mean logMAR BCVA in three groups were 0, 0, and 0, respectively. It did not show any difference. No abnormal symptoms occurred in the eyes included in our study. There was no statistical difference in the retinal thickness of the fovea and FAZ related parameters. The reduction in deep vascular density may be described as subclinical retinal microvascular ischemia in this study. Sioufi et al. reported a slight reduction in deep capillary density in both treated eyes in patients with bilateral RB and fellow eyes in patients with unilateral RB after IVC at a mean 10-year follow-up (13). Yi et al. showed that significant changes in vessel density were detected in patients with bilateral RB, but not in unilateral RB (12). In our study, there were no statistically significant differences in the vascular density between group I (IAC) and group II (IAC + IVC). In the rabbit experiments conducted by Daniels et al. the complete chemotherapy dose injected into the OA eventually entered the blood supply through the systemic circulation. Although in unilateral IAC treatment, the drug can enter the contralateral eye through blood circulation and produce retinal toxicity (14). Our study suggested that the retinal microvasculature of the healthy fellow eyes could be affected by IAC. DCP represents a deep slab extending from the inner plexiform layer to the outer border of the outer plexiform layer. Each radial peripapillary capillaries was supplied by the capillaries from the superficial vascular plexus and drained to the intermediate plexus or deep vascular plexus. The oxygen saturation in the deep vascular plexus was lower than that in the superficial vascular plexus due to the vascular circulation. In addition, the deep vascular plexus may participate in vascular inflammation (15). Therefore, with toxicity, the DCP is likely to be affected more than the SCP.

The thinning of the submacular choroidal thickness and choroidal atrophy were observed in IAC treated eyes by spectral-domain optical coherence tomography (16). A report indicated that the incidence of choroidal atrophy was as high as 10–15% (17). Yi et al. found a slight reduction in retinal thickness in bilateral RB eyes treated with IVC (12). There was a statistical difference between eyes with and without receiving IAC + IVC treatments in the thickness of the OPL-BRM in the fovea in our study. The fovea thickness including the full, the outer, the ILM-BRM, and the ISOS-BRM also tended to decrease in patients with IAC + IVC, which was worth consideration. The retinal arterial occlusion was also observed in clinical practice (18, 19). The blood flow velocity in the posterior ciliary arteries and central retinal arteries was assessed to decrease after IAC by color Doppler imaging (20). Tse et al. guessed that the choroidal atrophy was caused by the damage of the posterior ciliary arteries in a patient who had enophthalmos and choroidal atrophy after IAC (21).

We considered that the effect on vascular density and retinal thickness may resulted from the chemotherapy. In a research on the IAC rabbit model, melphalan seemed to be dose-dependently linked to retinal toxicities (22). The drug deposition could be one of the key reasons. In five enucleated eyes, Eagle et al. identified intravascular birefringent foreign material in occluded vessels by histopathologic observations (23). Tse et al. searched the ocular and orbital histopathology of the Macaca mulatta and found that leukostasis deposition and damaged endothelial cell in retinal arteriole occlusion (24). Steinle et al. reported the change in the vascular endothelium and monocytes caused by chemotherapeutic drugs *in vitro* and *in vivo*, suggesting that IAC induced vessel toxicities by stimulating an inflammatory response (25). These findings illustrated that chemotherapy drugs can cause severe toxicity to the retinal vessels.

There are a few important limitations to this study that warrant discussion. Firstly, our sample size was too small to definitively verify a statistical difference among the three groups in the retinal thickness. Secondly, the internal environment of RB patients cannot be completely ruled out. Ectopic S-phase and high levels of p53-independent apoptosis were detected in RB-deficient retinas, particularly in the differentiating retinal ganglion cell layer. Loss of RB resulted in more extensive retinal apoptosis during the postnatal retinal development. The retina in adults showed loss of photoreceptors and bipolar cells (26). Further self-controlled studies before and after treatments will be conducted to reduce individual differences including the tumor effects. Lastly, we did not enroll patients with only IVC treatments due to the small sample. The effects on fellow eyes before and after receiving IVC, whether it was different from IAC, and the synergism of IVC and IAC were still unclear. Future studies are needed to explore.

## Conclusion

In summary, our results have suggested a slight reduction in the DCP without measurable visual impairment in fellow eyes after IAC or IAC + IVC for unilateral RB. It is necessary to focus on long-term safety of different chemotherapy regimens and to determine the clinical significance of these findings in the future studies with a larger sample.

## Data availability statement

The original contributions presented in this study are included in the article/supplementary material, further inquiries can be directed to the corresponding author.

## Ethics statement

The studies involving human participants were reviewed and approved by the Ethics Review Board of Affiliated Eye Hospital of Wenzhou Medical University. Written informed consent to participate in this study was provided by the participants’ legal guardian/next of kin.

## Author contributions

LS designed, supervised the study, and revised the manuscript. YC, JM, and ZX analyzed data and wrote the manuscript. ZZ and SZ evaluated the clinical characteristics of the enrolled patients. JM, YC, SZ, and SW performed the research. All authors contributed to the article and approved the submitted version.
